# Supplementary material for: Single-cell mass cytometry on peripheral blood identifies immune cell subsets associated with primary biliary cholangitis
Source: Sci Rep. 2020 Jul 28;10:12584. doi: 10.1038/s41598-020-69358-4 (PMC7387528; doi:10.1038/s41598-020-69358-4)
Supplement: Supplementary file 1 — Supplementary information. [file 41598_2020_69358_MOESM1_ESM.docx]

**Supplementary Information for**

**Single-Cell Mass Cytometry on Peripheral Blood Identifies Immune Cell Subsets Associated with Primary Biliary Cholangitis**

Jin Sung Jang^1,2^, Brian Juran^3^, Kevin Y. Cunningham^4,5^, Vinod K. Gupta^6,7^, Young Min Son^8,9^, Ju Dong Yang^10^, Ahmad H. Ali^3^, Elizabeth Ann L. Enninga^9,11^, Jaeyun Sung^6,7,12,*^, and Konstantinos N. Lazaridis^3,*,Ψ^

^1^Medical Genome Facility, Center for Individualized Medicine, Mayo Clinic, Rochester, MN, USA;

^2^Department of Laboratory Medicine and Pathology, Mayo Clinic, Rochester, MN, USA; ^3^Division of Gastroenterology and Hepatology, Department of Internal Medicine, Mayo Clinic, Rochester, MN, USA; ^4^Graduate Research Education Program (GREP), Mayo Clinic, Rochester, MN, USA; ^5^Department of Computer Science and Engineering, University of Minnesota Twin-Cities, Minneapolis, MN, USA; ^6^Microbiome Program, Center for Individualized Medicine, Mayo Clinic, Rochester, MN, USA; ^7^Division of Surgical Research, Department of Surgery, Mayo Clinic, Rochester, MN, USA; ^8^Division of Pulmonary and Critical Care Medicine, Department of Medicine, Mayo Clinic, MN, USA; ^9^Department of Immunology, Mayo Clinic, Rochester, MN, USA; ^10^Division of Digestive and Liver Diseases, Department of Medicine, Cedars Sinai Medical Center, Los Angeles, CA, USA; ^11^Department of Obstetrics and Gynecology, Mayo Clinic, Rochester, MN, USA; ^12^Division of Rheumatology, Department of Medicine, Mayo Clinic, Rochester, MN, USA

**^*^Corresponding Authors:**

Jaeyun Sung, PhD

Division of Surgical Research, Department of Surgery, Mayo Clinic, 200 First Street SW, Rochester, MN 55905 ([Sung.Jaeyun@mayo.edu](mailto:Sung.Jaeyun@mayo.edu))

Konstantinos N. Lazaridis, MD

Division of Gastroenterology and Hepatology, Department of Internal Medicine, Mayo Clinic, 200 First Street SW, Rochester, MN 55905 ([Lazaridis.Konstantinos@mayo.edu](mailto:Lazaridis.Konstantinos@mayo.edu))

**^Ψ^ Lead Contact**

| Supplementary Table S1. List of primary antibodies used in this study. | | | | | |
| --- | --- | --- | --- | --- | --- |
| No. | Species | Clone | Metal | Target | Amount required per test (μL) |
| 1 | Human | 3G8 | 148Nd | CD16 | 0.125 |
| 2 | Human | SK1 | 168Er | CD8a | 0.125 |
| 3 | Human | 2H7 | 171Yb | CD20 | 0.125 |
| 4 | Human | SK3 | 174Yb | CD4 | 0.125 |
| 5 | Human | HI30 | 089Y | CD45 | 0.250 |
| 6 | Human | HIB19 | 142Nd | CD19 | 0.250 |
| 7 | Human | IA6-2 | 146Nd | IgD | 0.250 |
| 8 | Human | Bu15 | 147Sm | CD11c | 0.250 |
| 9 | Human | 11F2 | 152Sm | TCRgd | 0.250 |
| 10 | Human | UCHT1 | 154Sm | CD3 | 0.250 |
| 11 | Human | HI100 | 155Gd | CD45RA | 0.250 |
| 12 | Human | L128 | 158Gd | CD27 | 0.250 |
| 13 | Human | L243 | 173Yb | HLA-DR | 0.250 |
| 14 | Human | G034E3 | 141Pr | CD196 (CCR6) | 0.500 |
| 15 | Human | A019D5 | 143Nd | CD127 (IL-7R) | 0.500 |
| 16 | Human | HIT2 | 144Nd | CD38 | 0.500 |
| 17 | Human | L291h4 | 149Sm | CD194 (CCR4) | 0.500 |
| 18 | Human | 6H6 | 151Eu | CD123 (IL-3R) | 0.500 |
| 19 | Human | RF8B2 | 153Eu | CD185 (CXCR5) | 0.500 |
| 20 | Human | CD28.2 | 160Gd | CD28 | 0.500 |
| 21 | Human | G025H7 | 163Dy | CD183 (CXCR3) | 0.500 |
| 22 | Human | UCHL1 | 165Ho | CD45RO | 0.500 |
| 23 | Human | ML5 | 166Er | CD24 | 0.500 |
| 24 | Human | G043H7 | 167Er | CD197 (CCR7) | 0.500 |
| 25 | Human | 2A3 | 169Tm | CD25 (IL-2R) | 0.500 |
| 26 | Human | M5E2 | 175Lu | CD14 | 0.500 |
| 27 | Human | NCAM16.2 | 176Yb | CD56 (NCAM) | 0.500 |
| 28 | Human | 80H3 | 162Dy | CD66b | 1.000 |
| 29 | Human | HP-3G10 | 164Dy | CD161 | 1.000 |
| 30 | Human | 11C3C65 | 150Nd | LAG-3 | 1.000 |
| 31 | Human | 29E.2A3 | 156Gd | PD-L1 | 1.000 |
| 32 | Human | 14D3 | 170Er | CTLA-4 | 1.000 |
| 33 | Human | 2A9-1 | 172Yb | CX3CR1 | 1.000 |
| 34 | Human | HI111 | 145Nd | CD11a | 0.250 |
| 35 | Human | F38-2E2 | 159Tb | Tim-3 | 1.000 |
| 36 | Human | EH12.2H7 | 161Dy | PD-1 | 1.000 |

| Supplementary Table S2. Distribution of cell counts from all 66 PBC and control samples across three different stages of the mass cytometry experiment. | | | | | | | | | |
| --- | --- | --- | --- | --- | --- | --- | --- | --- | --- |
| Group | Sex | Age^†^ (yr) | Age dx^‡^ (yr) | Histological stage at biopsy^§^ | Age of sample* (yr) | Post-thaw cell count | Viability (%) | Post-deconvolution event count | Post-QC event count |
| PBC_1 | F | 50 | 47 | N/A | 13 | 23,031,054 | 1.2 | 202,003 | 65,733 |
| PBC_2 | F | 50 | 47 | 3 | 13 | 6,773,839 | 76.2 | 186,356 | 55,738 |
| PBC_3 | F | 51 | 47 | 1 | 12 | 7,436,561 | 83.7 | 456,148 | 246,364 |
| PBC_4 | F | 51 | 51 | 2 | 9 | 11,107,924 | 77.2 | 132,597 | 66,214 |
| PBC_5 | F | 52 | 41 | 1 | 14 | 4,961,617 | 88.2 | 294,370 | 215,064 |
| PBC_6 | F | 53 | 51 | 1 | 10 | 3,847,306 | 82.6 | 168,051 | 105,193 |
| PBC_7 | F | 54 | 45 | 3 | 15 | 4,070,168 | 85.0 | 144,518 | 87,427 |
| PBC_8 | F | 55 | 52 | 2 | 13 | 7,890,000 | 77.0 | 195,881 | 115,118 |
| PBC_9 | F | 56 | 54 | N/A | 13 | 6,380,000 | 93.0 | 411,839 | 290,066 |
| PBC_10 | F | 56 | 48 | 3 | 11 | 9,850,000 | 72.7 | 142,290 | 48,431 |
| PBC_11 | F | 56 | 45 | 1 | 13 | 5,330,000 | 91.7 | 273,194 | 167,477 |
| PBC_12 | F | 56 | 49 | 2 | 13 | 5,410,000 | 87.6 | 234,168 | 159,360 |
| PBC_13 | F | 57 | 53 | 2 | 13 | 7,210,000 | 79.9 | 175,132 | 115,964 |
| PBC_14 | F | 57 | 56 | N/A | 12 | 6,590,000 | 82.2 | 115,846 | 60,835 |
| PBC_15 | F | 58 | 43 | 2 | 11 | 5,860,000 | 85.2 | 48,225 | 29,516 |
| PBC_16 | F | 58 | 46 | 3 | 15 | 1,520,000 | 88.5 | 33,315 | 17,639 |
| PBC_17 | F | 59 | 58 | 2 | 8 | 9,160,000 | 75.8 | 69,586 | 39,353 |
| PBC_18 | F | 59 | 54 | 1 | 14 | 1,580,000 | 78.1 | 35,608 | 18,814 |
| PBC_19 | F | 59 | 55 | 2 | 13 | 6,000,000 | 77.7 | 91,287 | 41,036 |
| PBC_20 | F | 59 | 55 | 3 | 13 | 8,730,000 | 81.8 | 33,363 | 17,665 |
| PBC_21 | F | 59 | 50 | N/A | 9 | 5,600,880 | 80.8 | 599,286 | 397,133 |
| PBC_22 | F | 60 | 37 | 3 | 11 | 3,589,255 | 82.8 | 292,122 | 178,702 |
| PBC_23 | F | 60 | 54 | 1 | 15 | 6,474,735 | 81.5 | 353,941 | 249,787 |
| PBC_24 | F | 60 | 56 | 1 | 11 | 14,568,153 | 4.7 | 103,234 | 53,999 |
| PBC_25 | F | 60 | 55 | 3 | 8 | 3,190,449 | 78.1 | 394,524 | 238,170 |
| PBC_26 | F | 60 | 57 | N/A | 8 | 7,213,699 | 81.2 | 422,794 | 299,032 |
| PBC_27 | F | 62 | 62 | 2 | 10 | 8,767,870 | 78.1 | 367,743 | 244,045 |
| PBC_28 | F | 62 | 39 | N/A | 14 | 2,228,623 | 94.7 | 281,392 | 156,677 |
| PBC_29 | F | 62 | 49 | 4 | 12 | 4,504,163 | 81.1 | 661,733 | 430,210 |
| PBC_30 | F | 63 | 43 | 3 | 14 | 2,797,508 | 88.1 | 515,905 | 340,659 |
| PBC_31 | F | 64 | 60 | N/A | 13 | 6,545,112 | 71.8 | 246,500 | 127,622 |
| PBC_32 | F | 64 | 63 | 4 | 11 | 7,207,834 | 74.6 | 366,443 | 218,469 |
| PBC_33 | F | 65 | 48 | 2 | 11 | 16,175,108 | 87.3 | 543,394 | 401,510 |
| Control_1 | F | 51 | _ | _ | 12 | 13,377,600 | 72.9 | 125,621 | 33,100 |
| Control_2 | F | 51 | _ | _ | 13 | 5,935,174 | 86.2 | 523,610 | 189,651 |
| Control_3 | F | 50 | _ | _ | 11 | 8,879,301 | 74.3 | 285,820 | 134,048 |
| Control_4 | F | 51 | _ | _ | 11 | 10,263,393 | 71.1 | 90,463 | 29,730 |
| Control_5 | F | 52 | _ | _ | 13 | 4,433,786 | 80.8 | 157,192 | 95,262 |
| Control_6 | F | 53 | _ | _ | 9 | 5,606,745 | 81.9 | 262,079 | 171,887 |
| Control_7 | F | 54 | _ | _ | 13 | 7,348,589 | 74.7 | 127,357 | 56,332 |
| Control_8 | F | 55 | _ | _ | 13 | 11,700,000 | 73.8 | 183,802 | 76,000 |
| Control_9 | F | 56 | _ | _ | 12 | 4,450,000 | 82.1 | 352,184 | 184,687 |
| Control_10 | F | 56 | _ | _ | 11 | 11,700,000 | 76.7 | 176,555 | 84,522 |
| Control_11 | F | 56 | _ | _ | 12 | 1,960,000 | 92.8 | 187,868 | 105,938 |
| Control_12 | F | 57 | _ | _ | 13 | 10,300,000 | 55.2 | 279,858 | 54,553 |
| Control_13 | F | 57 | _ | _ | 13 | 6,840,000 | 72.1 | 141,117 | 83,531 |
| Control_14 | F | 57 | _ | _ | 11 | 5,750,000 | 87.3 | 66,461 | 33,955 |
| Control_15 | F | 58 | _ | _ | 12 | 9,880,000 | 65.0 | 83,576 | 34,763 |
| Control_16 | F | 58 | _ | _ | 13 | 4,250,000 | 86.6 | 258,465 | 178,920 |
| Control_17 | F | 59 | _ | _ | 9 | 1,920,000 | 89.9 | 109,464 | 70,916 |
| Control_18 | F | 59 | _ | _ | 13 | 5,230,000 | 84.9 | 136,440 | 90,915 |
| Control_19 | F | 59 | _ | _ | 12 | 7,880,000 | 85.8 | 47,925 | 25,753 |
| Control_20 | F | 59 | _ | _ | 13 | 5,120,000 | 87.2 | 111,198 | 71,984 |
| Control_21 | F | 59 | _ | _ | 10 | 1,735,980 | 94.9 | 231,317 | 143,649 |
| Control_22 | F | 60 | _ | _ | 11 | 3,612,714 | 96.8 | 847,255 | 510,051 |
| Control_23 | F | 60 | _ | _ | 12 | 7,841,232 | 82.2 | 523,957 | 381,778 |
| Control_24 | F | 60 | _ | _ | 9 | 6,744,515 | 62.7 | 213,287 | 117,966 |
| Control_25 | F | 60 | _ | _ | 9 | 4,111,222 | 96.4 | 674,103 | 485,811 |
| Control_26 | F | 61 | _ | _ | 9 | 4,240,248 | 95.6 | 644,313 | 477,152 |
| Control_27 | F | 62 | _ | _ | 9 | 5,624,339 | 88.6 | 509,045 | 346,213 |
| Control_28 | F | 62 | _ | _ | 13 | 2,867,885 | 90.4 | 338,518 | 212,315 |
| Control_29 | F | 62 | _ | _ | 12 | 4,744,620 | 84.5 | 351,794 | 187,192 |
| Control_30 | F | 63 | _ | _ | 13 | 4,375,138 | 79.6 | 640,654 | 445,053 |
| Control_31 | F | 63 | _ | _ | 13 | 6,433,681 | 79.9 | 428,364 | 299,692 |
| Control_32 | F | 64 | _ | _ | 11 | 6,052,470 | 93.8 | 801,474 | 477,574 |
| Control_33 | F | 65 | _ | _ | 12 | 6,087,658 | 77.9 | 338,325 | 216,743 |
| ^†^Age of subject at blood sample collection. ^‡^Age of patient at PBC diagnosis. ^§^Histological stage at first biopsy. Histological data was available for 26 patients only. *Number of years the sample was stored prior to preparation for immunophenotyping analysis.  Abbreviations: N/A, not available. | | | | | | | | | |

| Supplementary Table S3. Clinical, Imaging, histological, and endoscopic features of the PBC patients who developed liver cirrhosis and portal hypertension (n = 7). | | | | | | | | | | |
| --- | --- | --- | --- | --- | --- | --- | --- | --- | --- | --- |
| Case # | Year of PBC diagnosis | Liver biopsy | Year of liver biopsy | Histological stage | Date of cirrhosis diagnosis | Parenchymal changes on imaging^†^ | Varices | Splenomegaly | Ascites | Encephalopathy |
| 1 | 1991 | Yes | 1991 | 3 | 2002 | Yes | Yes | Yes | No | Yes |
| 2 | 1980 | Yes | 2003 | 4 | 2003 | Yes | Yes | Yes | No | Yes |
| 3 | 1997 | Yes | 1997 | 2 | 2001 | Yes | Yes | Yes | Yes | Yes |
| 4 | 2006 | Yes | 2006 | 4 | 2006 | Yes | Yes | Yes | Yes | No |
| 5 | 2000 | Yes | 2000 | 3 | 2005 | Yes | Yes | Yes | Yes | No |
| 6 | 2006 | Yes | 2006 | 3 | 2006 | Yes | Yes | Yes | Yes | No |
| 7 | 1993 | Yes | 1998 | 4 | 1998 | Yes | Yes | Yes | No | No |
| ^†^Imaging features characteristic of cirrhosis: Liver surface nodularity and decreased liver size. | | | | | | | | | | |

**
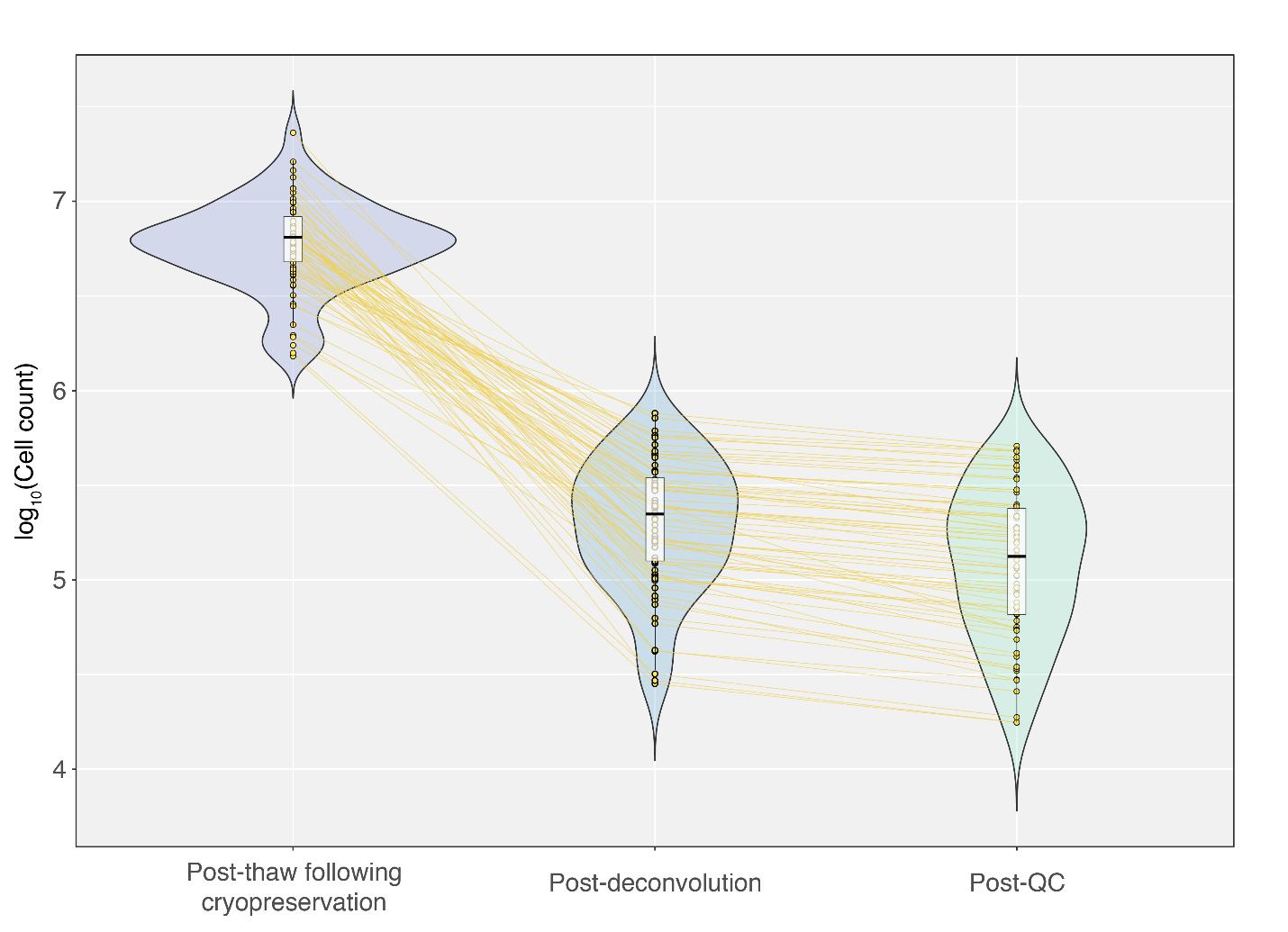
**

**Supplementary Figure S1.** Distribution of cell counts from all 66 PBC and control samples across three different stages of the mass cytometry experiment. Gold lines connect points corresponding to the same sample. Post-thaw following cryopreservation: The number of viable and non-viable cells counted after thawing following cryopreservation (or the original number of cells that were cyropreserved). These numbers corresponds to the total yield of cells before metal-conjugated antibody labeling; Post-deconvolution: The number of events in each sample after antibody labeling and sample debarcoding (i.e., deconvolution); Post-QC: The number of events in each sample after the quality-control process (which includes elimination of debris, dead cells, and doublets) indicating the actual number of viable cells that were profiled.

**
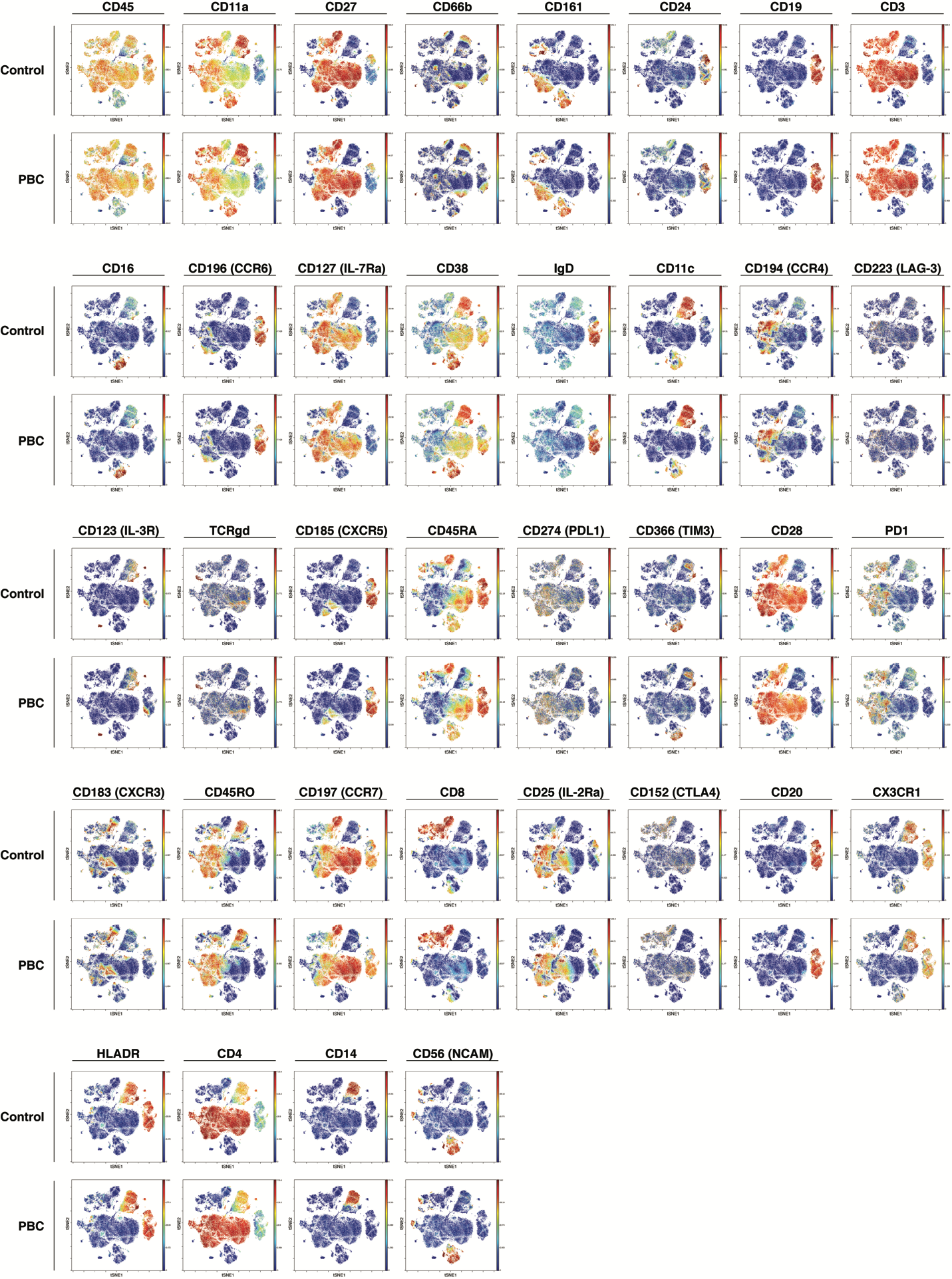
**

**Supplementary Figure S2.** t-SNE maps colored by channel intensities show relative expression of immune cell markers. Mass cytometry samples from 33 PBC patients were concatenated into 100,000 randomly-sampled total events and mapped onto a t-SNE plot using viSNE (PBC). Analogously, samples from 33 age-/sex-matched controls were mapped using viSNE (Control). Each point in the t-SNE plot represents a single event (e.g., cell) detected by the mass cytometer, and colors vary according to the degree of marker expression. Observed regional differences in the viSNE maps correspond to differences in expression patterns of immune cell markers.


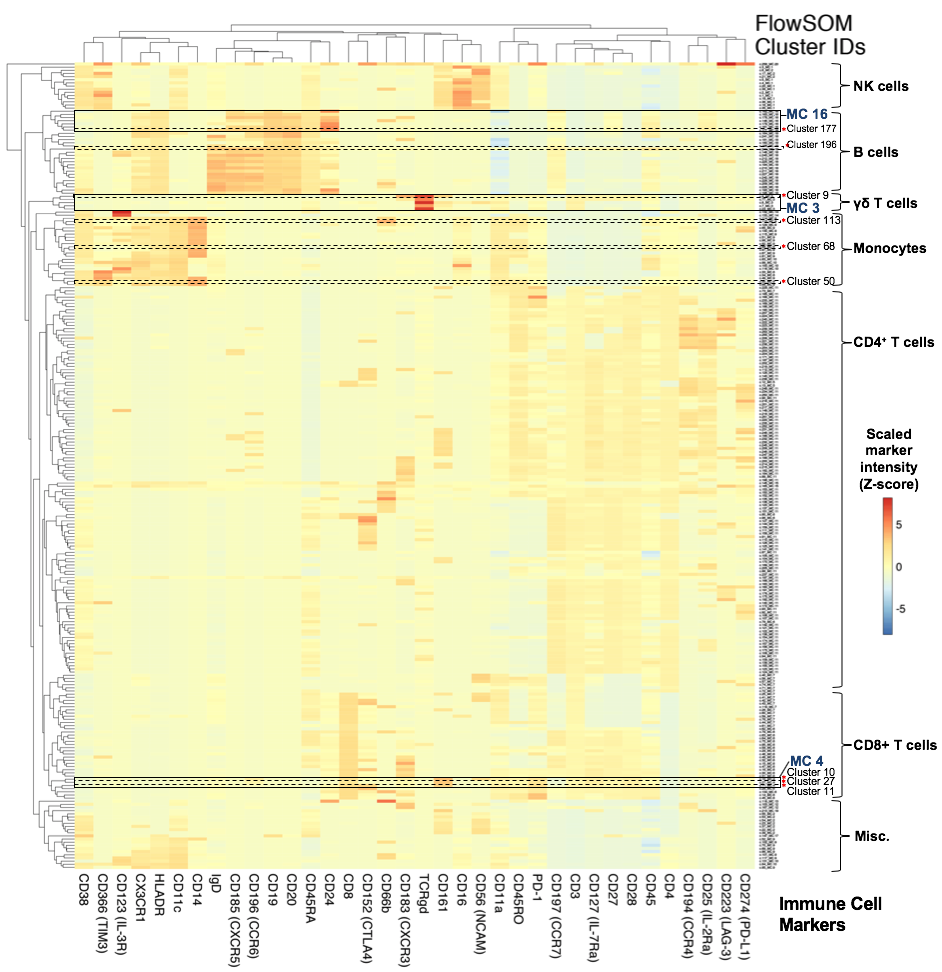


**Supplementary Figure S3.** Marker expression patterns for all 256 FlowSOM immune cell clusters show variation within metaclusters. The three meta-clusters associated with PBC (MC 3, MC 4 and MC 16) are highlighted by boxes with solid lines. The nine clusters that are independently associated with PBC are highlighted by boxes with dashed lines. Cell types (i.e., NK cells, B cells, etc.) were determined by visual inspection of marker expression and are meant to be illustrative, not a comprehensive assessment.

**
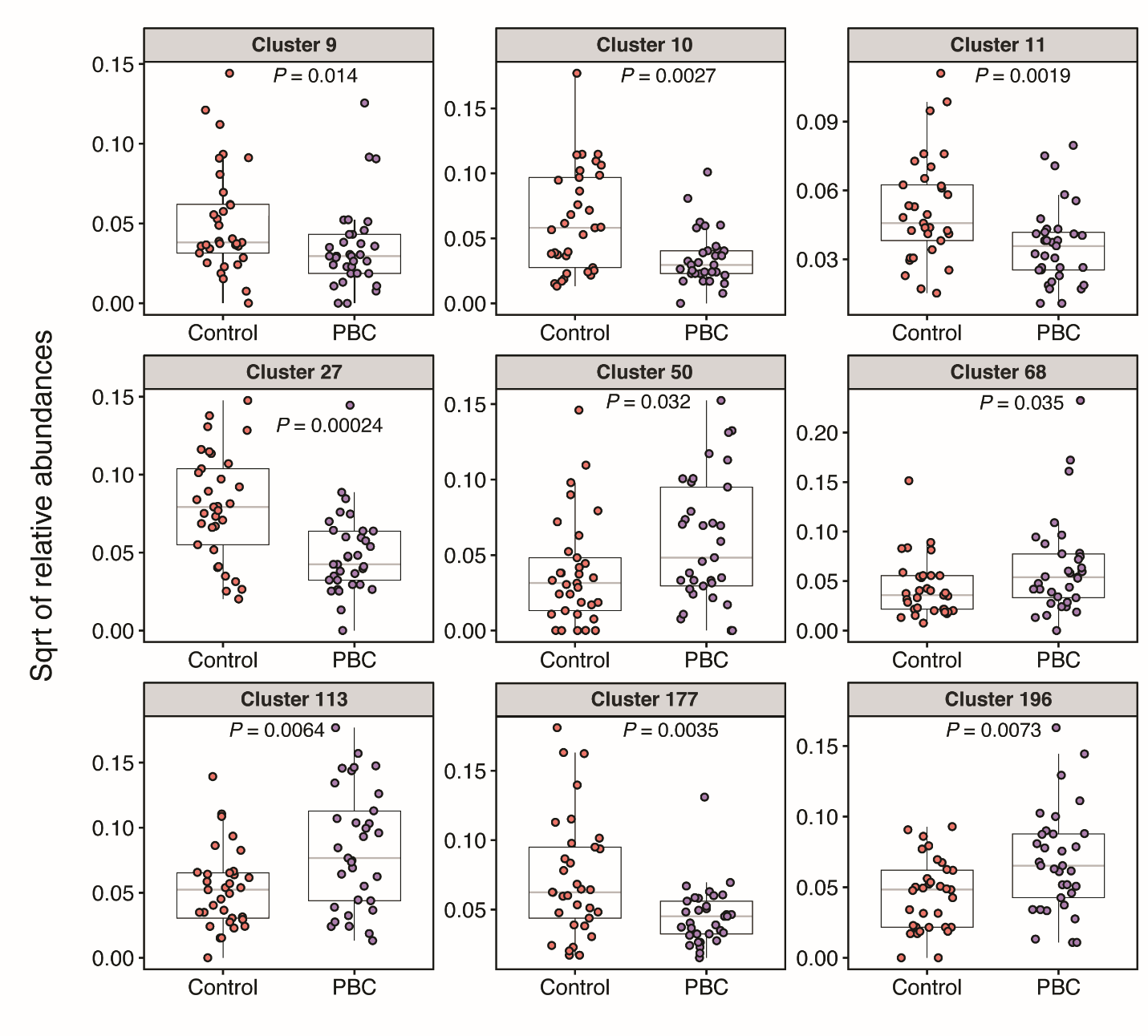
**

**Supplementary Figure S4.** FlowSOM clusters whose relative abundances were significantly different between PBC and control (fold-change≥2.0 and *p*<0.05 based on Mann-Whitney *U* test).

**
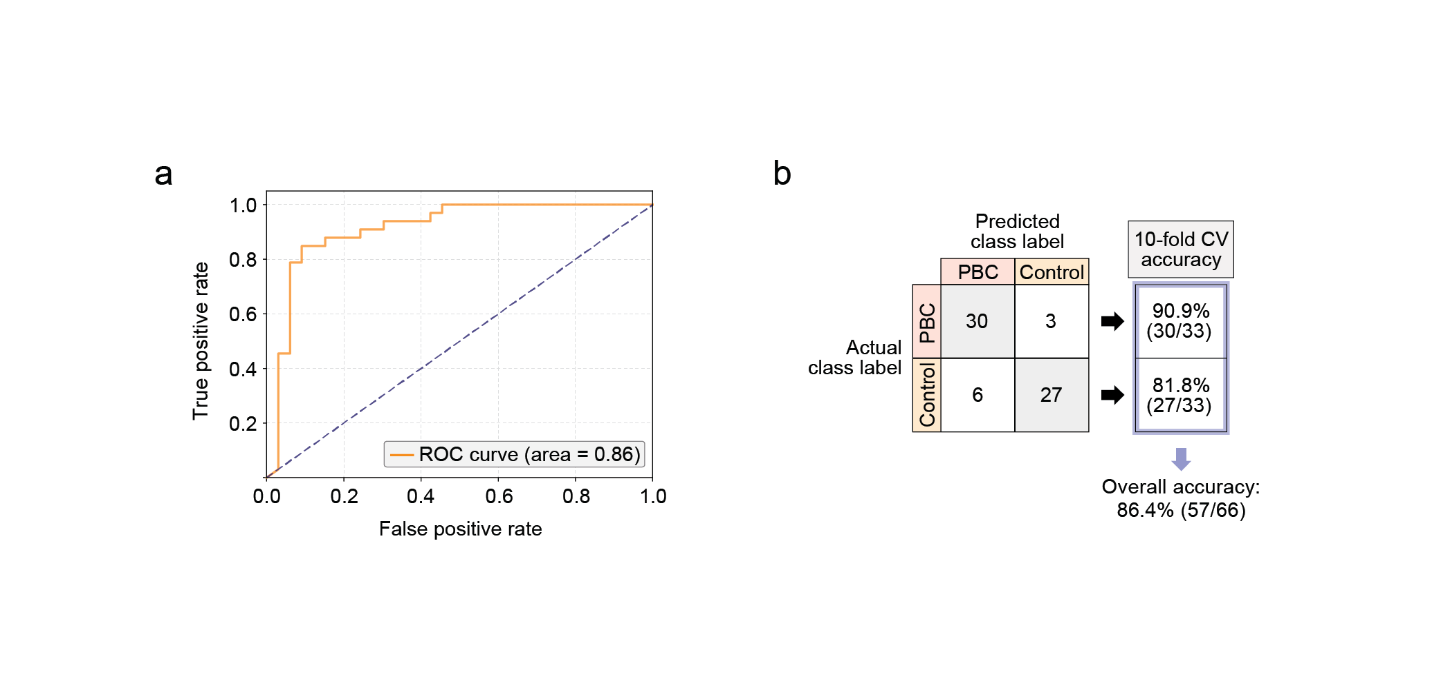
**

**Supplementary Figure S5.** A neural network-based classification model, which was trained upon FlowSOM cluster features and class labels, was able to distinguish immune profiles of PBC from those of control with high prediction accuracy. Model performance was evaluated in ten-fold cross-validation. (a) ROC curve with an AUC of 0.86; and (b) confusion matrix of model predictions, demonstrating an overall phenotype prediction accuracy of 86.4% and group-wise accuracies of 90.9% and 81.8% for PBC and control, respectively.
